# Knowledge, attitudes and practices around health research: the perspective of physicians-in-training in Pakistan

**DOI:** 10.1186/1472-6920-9-46

**Published:** 2009-07-17

**Authors:** Hassan Khan, Sadaf Khan, Arshad Iqbal

**Affiliations:** 1 Department of Surgery, Aga Khan University, Karachi, Pakistan; 2 Medical College, Aga Khan University, Karachi, Pakistan

## Abstract

**Background:**

Health research training is an essential component of medical education and a vital exercise to help develop physician research skills. This study was carried out to assess the level of knowledge, attitudes and practices towards research amongst a group of Post Graduate Medical Trainees (PGMTs') at Aga Khan University (AKU), Pakistan.

**Methods:**

A cross sectional health research survey was carried out on all PGMTs' at AKU Pakistan. AKU is a tertiary care health facility which offers residency in 28 specialties and fellowship in 16 programs. Knowledge, attitudes and practices related to health research were assessed using a pretested, structured and validated questionnaire. Health research related practices of the residents were examined using questions graded on Likert scale.

**Results:**

Mean percentage score ± SD on the knowledge scale was 36.9% ± 20.2 and 47.19% ± 25.18 on the attitude scale. Of 104(55.6%) who had previously participated in research 28(26.9%) had been involved in basic science research only, 62(59.6%) in clinical research and 14(13.5%) had participated in both clinical and basic science research projects. 88(47.1%) planned to pursue a future research career. Those who planned to pursue a future research career had more positive health research attitudes p < 0.001. Limited time (45%), poor research infrastructure (20%) and inadequate research funding opportunities (20%) were the major hurdles faced by PGMTs' to pursue research.

**Conclusion:**

PGMTs' demonstrate inadequate knowledge, while they have moderate attitudes towards health research. Residency training and research facilities at the institution need to undergo major transformation in order to encourage meaningful research by resident trainees.

## Background

Research experience is invaluable to the physician's evidence-based practice as it imparts skills such as literature search, collecting and analyzing data and critical appraisal of evidence [1-3]. Training for research skills and experience of research early in career has been associated with continued professional academic work and may help inform residents' career decisions[4].

Research training is currently being incorporated as part of medical school curricula and residency training programs to build a task force of competent physician scientists. The motto of medical education is to prepare physicians to meet the challenges of practice by fulfilling their roles of clinicians, educators and clinical researchers. In order to evaluate whether efforts and interventions to promote research are paying off, we need to assess the level of research knowledge, attitudes and practices of residents. It will also help identifying difficulties and challenges faced by them whilst pursuing research during residency, and thus allow us to build a research-facilitating curricula and environment in residency programs.

In Pakistan, medical schools offer a 5 year program leading to an MB; BS (Bachelors of Medicine; Bachelors of Surgery) degree. Basic health sciences are the primary focus of instruction during the first two years, with gradually increasing exposure to clinical disciplines over the next three years. After graduation from medical school all doctors are required to do a year of internship, which is followed by a residency training program of their choice.

The primary objectives of our study were to asses the existing level of knowledge and attitudes towards health research amongst post graduate medical trainees and to determine their research involvement and practices.

## Methods

A cross-sectional descriptive study was conducted on interns and residents at the Aga Khan University Hospital (AKUH), Karachi, Pakistan. The university hospital which is a tertiary care facility attracts residents from all parts of the country.

The AKUH offers residency in 28 specialties. All programs are overseen by the Postgraduate Medical Education (PGME) committee at AKU, which sets common goals and objectives for the trainees or residents.

### Study sampling

At the time of study, a total of 339 residents and 70 interns were enrolled at the university hospital. We required a sample size of 218 subjects to fulfill the objectives of our study at a 95% confidence level. This sample size was calculated assuming a 50% prevalence of good knowledge and attitude, 5% bound-on error, and 10% non-response rate. The subjects were selected among interns and residents using convenience sampling.

The principle investigator and medical student involved in the study went to all departments to distribute the self-administered questionnaire amongst the residents after seeking their verbal consent. The resident was requested to fill the questionnaire if he could spare his time. Other wise, the resident's pager number was noted and he/she was approached at a later time.

### Questionnaire

The information was collected on a pre-tested and structured questionnaire, adapted from the validated questionnaire designed by Vodopivec et al[5]. This was adapted after peer review. The questionnaire was then pre-tested on a group of residents who were expected to identify questions most valid in ascertaining our objectives. Further modifications were made to develop a final questionnaire. The questionnaire consisted of parts namely; resident profile, evaluation of knowledge and attitudes of health research, and research practices of the PGMTs'. Demographic details of subjects included age, gender, year of residency and mode of learning at medical school, Problem based learning (PBL) versus Lecture based learning (LBL). Medical School was categorized as private or government institution. Residency program was broadly divided into Medicine, Surgery, and Other specialty.

Knowledge was assessed by ten multiple-choice questions. For each respondent, the percentage of correct answers was calculated as a representative of knowledge score. Six questions were asked to assess the attitudes of trainees towards health research and each answer was scored on a scale of 0 (unfavorable attitude) to 1 (favorable attitude). For each individual, score of questions was summed and converted into percentage (0 to 100) to represent the attitude score. The Chronbach alpha for these six items of the attitude scale was 0.52. Research practices included questions regarding current published output, factors given importance to while publishing (responses recorded on 5 item Likert scale), future research plans and difficulties encountered in pursuing research.

The study was conducted in compliance with the "Ethical principles for medical research involving human subjects" section of the Helsinki Declaration. Verbal consent was taken from all participants before administration of questionnaire. A reference number was allocated to every subject to ensure confidentiality, and to be used instead of name.

### Statistical analysis

Data were entered and analyzed in Statistical Package for Social Sciences 15.0 (SPSS, Inc., Chicago, IL, USA) and Microsoft Excel (Microsoft Corp, Redmond, WA). Descriptive statistics were performed to determine the mean scores of various groups on the knowledge and attitude scales. ANOVA and t-test were used to look for putative associations of, mode of study in medical school, gender, specialty of residency program and the year of training with the knowledge and attitude scores.

## Results

Of the 218 post graduate trainees approached a total of 187(response rate 86%) returned the completed questionnaires and were included in the analysis. Of these 107(57,2%) were males and 80(42,8%) were females. Mean age of the sample was 27.0 ± 2.70 years. Mean percentage score ± SD on the knowledge scale was 36.85% ± 20.17 and 47.19% ± 25.18 on the attitude scale.

Proportion of subjects with correct answer for each question on the knowledge questionnaire is shown in Table 1. The responses of trainees to attitude questions are shown in Table 2. Out of 187 students 133(71.1%) felt confident in interpreting and writing a research paper, 119(64%) felt that they required assistance, while 14(7.5%) did not feel they needed assistance. Of 104(55.6%) who had previously participated in research 28(26.9%) had been involved in basic science research only, 62(59.6%) in clinical research and 14(13.5%) had participated in both clinical and basic science research projects. 88(47.1%) planned to pursue a career in research. Those who planned to pursue research in future had more positive health research attitude p < 0.001 but their score on the knowledge scale did not differ significantly from those who did not plan to pursue research in future.

**Table 1:** Proportion of PGMTS' with correct answers for questions

1. How would you define the scientific hypothesis?	62 (33.2)
a. A proposed idea or thought	
b. An answer or solution to a question	
c. An answer or solution to a question which has a capacity of verification or empirical demonstration*	
d. logical deduction of the premises that may or may not be verified empirically	
2. How would you define scientific theory?	56 (29.9)
a. Speculation or assumption with no or insufficient evidence	
b. Scientific hypotheses that may be proven, but lacking evidence for verification.	
c. Set of scientific knowledge on a given topic or area	
d. System of hypotheses logically connected to one another, with common background, some of which	
have been verified*	

3. How would you define the scientific truth?	33 (17.6)
a. The truth that will be reached through scientific research	
b. Absolute truth	
c. Consensus of competent experts *	
d. Fact that can be found in the textbooks	
e. Facts that your professors teach you	

4. The essential characteristic of science is:	49 (26.2)
a. All scientific conclusions are temporary*	
b. Scientific theory cannot merely explain natural phenomena, but must somehow also exert influence	
upon them	
c. Rather obvious scientific conclusion does not have to be testable	
d. An experiment is not an objective model of the nature but serves as an introduction into real	
research of natural phenomena	

5. A scale from 1 to 5 (like grades on an examination) is called:	106(56.7)
a. Ratio scale	
b. Nominal	
c. Ordinal *	
d. Interval	
e. It is not a scale	

6. Representativeness is a key characteristic of a:	80 42.8)
a. Scientific paper	
b. Professional paper	
c. Scientific research	
d. Sample*	
e. Population	

7. MEDLINE is:	73 (39)
a. The first and best known "on-line" medical journal	
b. International association of medical informaticians	
c. Printed form of the Excerpta Medica	
d. Abbreviation (acronym) that lists the parts of the research article	
e. Medical database*	

8. In the previous year, you have published a paper in a prestigious Journal of Immunology.	
Now you want to check the number of citations your paper has received.	
The best way to do it would be to search the:	62 (33.2)
a. author index of the MEDLINE database	
b. Corporate index of the Science Citation Index database	
c. Author index of the Current Contents database	
d. Citation index of the Science Citation Index database*	
e. Author index of the Science Citation Index database	

9. The part of a scientific paper is:	78 (41.7)
a. Author's curriculum vitae	
b. Letter to the editor enclosed with the paper	
c. Description of the timeline	
d. Acknowledgment to persons who assisted you during the research*	

10. All listed rules apply to the process of writing an Introduction section of a scientific paper EXCEPT:	90 (48.1)
a. clearly state why the research has been started	
b. do not explain textbook facts	
c. do not explain words from the title of the paper	
d. make it longer rather than shorter*	
e. clearly define the question to which your research aims to provide an answer	

Mean score (+ SD) 36.85 + 20.17

¶Questions used with permission of Vodopivec *et al.*, *Correct

**Table 2:** Attitudes of PGMTS' towards scientific research

**Statement**	**Yes**	**No**	**Undecided**
**1. Do you feel confident in interpreting and writing a research paper?**	133 (71.5%)	53 (28.5%)	1 (0.5%)

**2. Have you ever participated in a research project?**	104 (55.6%)	83 (44.4%)	0

**3. Have you ever written a scientific paper?**	61 (32.6%)	125 (66.8%)	1 (0.5%)

**4. Do you think PGMTs' should participate in research?**	134 (72%)	52 (28%)	1 (0.5%)

**5. Do you think PGMTs' can plan and conduct a research project and write a scientific paper?**	133 (71.7%)	16 (8.6%)	38 (20.3%)

**6. PGMTs' can plan and conduct research project without supervision**	122 (65.2%)	24 (12.8%)	41 (21.9%)

Table 3 shows the number of post graduate trainees in different groups with respect to gender, type of medical school, type of medical school curriculum, type and year of PGMT residency. Mean scores ± SD on knowledge and attitude scale are also compared. Males had better attitudes towards health research even though the difference on the knowledge scales was not significant. Type of medical school was a significant predictor of PGME trainee knowledge and attitudes towards research, with those from private medical schools scoring better significantly on both scales. Medical school curriculum was not seen to influence the scoring of PGMTs' on both scales. Surgical residents performed better on the attitudes scale than other two groups, though the difference in knowledge score between the groups was not significant. Year of post graduate training was not a significant factor in determining scores on both knowledge and attitude scales when evaluated through a multivariate linear regression model.

**Table 3:** PGMTS' knowledge and attitudes towards health research

			**Knowledge**		**Attitude**	
		No	Mean ± SD	p-value	Mean ± SD	p-value

**Gender**	Male	107	37.20 ± 20.73	0.782	51.48 ± 25.41	0.006
				
	Female	80	36.37 ± 19.50		41.46 ± 23.83	

**Medical School Type**	Government School	120	34.50 ± 18.37	0.045	43.19 ± 23.45	0.005
				
	Private School	67	41.05 ± 22.57		54.35 ± 26.72	

**Medical School Curriculum**	PBL	11	31.82 ± 19.40	0.395	57.58 ± 22.50	0.144
				
	LBL	176	37.16 ± 20.22		46.54 ± 24.25	

**Residency Programme**	Surgery	44	37.50 ± 18.57	0.609	54.36 ± 25.83	0.044
				
	Medicine	50	35.20 ± 20.83		41.83 ± 22.93	
				
	Other	22	32.27 ± 22.24		45.08 ± 24.22	

**PGMT year**	Intern	70	39.01 ± 20.08	0.283	47.18 ± 26.00	0.357
				
	1^st^	37	37.30 ± 21.17		45.05 ± 24.41	
				
	2^nd^	34	34.12 ± 22.58		46.08 ± 27.31	
				
	3^rd^	28	35.0 ± 19.15		45.24 ± 20.09	
				
	4^th^	10	34.0 ± 11.74		55.83 ± 24.23	
				
	5^th^	08	37.15 ± 20.59		59.53 ± 32.07	

**Figure 1:**
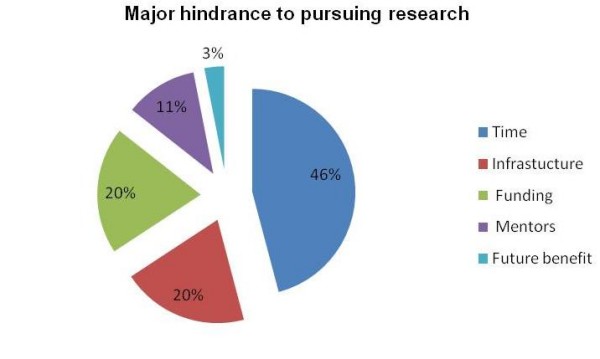
**Major hindrance cited by PGMTS' for pursuing research in Pakistan**.

In Figure 1 the responses of PGMTs' about the barriers faced by them to pursue research are summarized, which most commonly include lack of future benefit, time and resource constraints. Responses of all PGMTs' who had published at least one research manuscript (letter, case report, review or original article) regarding the factors they considered most important while selecting a journal to submit their manuscript, are summarized in Table 4.

**Table 4:** Factors PGMTS' (who have previously published manuscripts) consider important while selecting a journal to submit their research?

	**Greatest**	**Great**	**Neutral**	**Less**	**Least**	**Response Average**
**Open Access**	14(25.5%)	21(38.2%)	16(29.1%)	2(3.6%)	2(3.6%)	3.78

**Peer Review**	21(35.0%)	24(40.0%)	11(18.3%)	3(5.0%)	1(1.7%)	4.02

**Publishing Time**	21(34.4)	22(36.1%)	12(19.7%)	4(6.6%)	2(3.3%)	3.92

**Impact Factor**	24(40.0%)	23(38.3%)	11(18.3%)	1(1.7%)	1(1.7%)	4.13

**Acceptance Rate**	21(35.0%)	24(40.0%)	13(21.7%)	2(3.3%)	0(0%)	4.07

**Local Journal**	19(32.2%)	19(32.25)	14(23.7%)	4(6.8%)	3(5.1%)	3.80

**International Journal**	32(54.2%)	17(28.8%)	6(10.2%)	2(3.4%)	2(3.4%)	4.27

## Discussion

We report poor level of knowledge towards research (mean score 36.9%) amongst Pakistani post graduate trainees. About 80.2% of the trainees scored in the first two quartiles of knowledge score.

Better results were observed on the attitude score (mean 47%). Our findings are in dispute with our previous work amongst undergraduate medical students at Aga Khan University who had fared better on both knowledge (mean 49%) and attitude scale (mean 53.7%).[6]

Even though residents who were trained at private medical schools scored better on both knowledge and attitudes scale compared to residents from government run schools, their overall mean score on both knowledge and attitude scale was inadequate. These scores reflect grave inadequacies of health research training at medical schools across the country. Furthermore limited research activities, poor funding and lack of mandatory research assignments in government institutions leaves students desensitized to research and compounds the inadequate health research training at this level. A pilot survey reporting on the attitudes of PGMTs' towards research cited poor research training and awareness as two most important factors for poor research activity in the country[7].

Residents' knowledge and attitudes towards health research did not improve significantly with increasing years of training at the university hospital, in contrast to earlier trend of improving scores seen in medical students with year of medical education[6]. This underlines the shortcomings of the curricula in imparting research skills to residents. In Pakistan PGMTs' are required to submit a research dissertation to College of Physicians and Surgeons Pakistan (CPSP_Pakistan's residency and fellowship training accreditation body) in order to be eligible for fellowship examinations. The university hospital also arranges research skills workshop for interns and first year residents. The aim of these workshops is to introduce basic statistics and epidemiology to the trainees. However, no mandatory manuscript writing workshops, research projects or research thesis are part of curriculum.

Each PGME program has a regular schedule of academic activities throughout the year including Journal clubs'; Evidence based Medicine sessions and research presentations. However the lack of increase in research related knowledge as residents' progress through training is a cause of concern. Previous studies have shown that frequent journal club activity helps trainees stay abreast with current literature, improve knowledge of research methodology, biostatistics and impart critical thinking skills[8,9]. Effective journal club activity requires club existence to be over 2 years with over 50% attendance[9]. Failure to see an improvement in knowledge scores could be due to poor attendance and lack of teaching of critical reading skills in club activity. The same can be extrapolated to other such academic forums. HEC (higher education commission) in Pakistan has made over 20,000 journals freely accessible to over 250 public and private universities across the country[10]. All residency programs must make an effort to organize regular journal club activities with mandatory attendance along with special workshops in critical reading and manuscript writing.

Gender was not a significant predictor of knowledge about health research, though males scored higher significantly on the attitudes scale. Type of residency did not affect trainee knowledge score. Overall 71.5% of trainees felt confident in interpreting and writing a research manuscript, however only 7.5% claimed the ability to do so without assistance. Only 65(34.7%) of trainees had published at least one research manuscript. While choosing a journal for manuscript submission those who had previously published considered, International publications to be the most important factor whilst open access was considered to be the least decisive. Perceptions about international journals are that their circulation is greater making manuscript publication in these journals desirable. Trainees may not give considerable importance to their work being part of an open access journal as most journals in the country are freely available through the Higher Education Commission to both public and private universities and thus can be easily accessed by them and their peers.

Limited time was the most important factor cited by residents in not being able to engage in research. Residency is a period of intense clinical training, punctuated by post graduate examinations at various levels of training. The levels of stress and work are physically and mentally exhaustive for the trainee. On average residents in Pakistan work about 80 hours a week and no legislation defines the upper limit of working hours. A study from AKUH reported over 46% residents to be morbidly stressed while 55% were under mild stress[11,12]. In this climate of clinical training, research is difficult to pursue. Limited infrastructure and lack of research funding were the second most important factors for not being able to engage in research. In Pakistan public funding for research is limited. Whatever funding is available does not provide financial security to the individual. Thus, there is little incentive to pursue research. Further more dearth of academic liberty, poor funding, and uncertain career options influence poor research output and brain drain according to recent survey of Pakistani students sent abroad for doctoral training[13]. Consequently 52.4% of trainees did not plan to pursue clinical research in future, yet most 72% recognized that PGMTs should be actively involved in research.

## Limitations

AKU trains medical graduates from medical schools across the country and offers very attractive and highly specialized training programs; it cannot however serve as a true representative of all the post graduate programs across the country. Since the trainees originate from different social and educational backgrounds our findings represent the impact of medical school training on research skills and knowledge of medical school graduates across the country. The use of a validated questionnaire allowed us to compare our findings with other studies and previous work done by us amongst medical students. However a low value of Crohnbach alpha < 0.7 for the attitude scale limits the reliability of results.

As this was a cross-sectional survey the study did not allow causative conclusions and convenience sampling further limits us from quantifying the error in extrapolating results to the entire population of PGMTs' in the country. Unfortunately we did not have sufficient numbers in the PBL group to see the effect of mode of learning at medical school on post graduate research activity. In addition Likert responses are prone to central tendency bias (respondents try to avoid extreme statements) and acquiescence bias (tend to agree with the presented statements). We recommend further detailed research to be carried out at the national level to evaluate the issue of PGMT research.

## Conclusion

In conclusion we report inadequate knowledge and moderate health research attitudes amongst PGMTs' which did not improve with year of training. This is a cause for much concern. It leaves medical education planners to ponder about the shortcomings of post graduate medical curricula in the country. These must be adapted to better impart the necessary research skills required of a 21^st ^century physician.

## Competing interests

The authors declare that they have no competing interests.

## Authors' contributions

HK conceived and designed the study. HK and AI administered the questionnaires. HK, SK, AI managed, analyzed and interpreted the data. HK and SK prepared the manuscript. All authors approved the final manuscript.

## Pre-publication history

The pre-publication history for this paper can be accessed here:



## References

[B1] Goodman NW (1994). Does research make better doctors?. Lancet.

[B2] Potti A, Mariani P, Saeed M, Smego RA (2003). Residents as researchers: expectations, requirements, and productivity. Am J Med.

[B3] Mylonakis E, Koutkia P (1999). The realities of resident research requirements. Jama.

[B4] Aslam F, Shakir M, Qayyum MA (2005). Why medical students are crucial to the future of research in South Asia. PLoS Med.

[B5] Vodopivec I, Vujaklija A, Hrabak M, Lukic IK, Marusic A, Marusic M (2002). Knowledge about and attitude towards science of first year medical students. Croat Med J.

[B6] Khan H, Khawaja MR, Waheed A, Rauf MA, Fatmi Z (2006). Knowledge and attitudes about health research amongst a group of Pakistani medical students. BMC Med Educ.

[B7] Aslam F, Qayyum MA, Mahmud H, Qasim R, Haque IU (2004). Attitudes and practices of postgraduate medical trainees towards research – a snapshot from Faisalabad. J Pak Med Assoc.

[B8] Kellum JA, Rieker JP, Power M, Powner DJ (2000). Teaching critical appraisal during critical care fellowship training: a foundation for evidence-based critical care medicine. Crit Care Med.

[B9] Akhund S, Kadir MM (2006). Do community medicine residency trainees learn through journal club? An experience from a developing country. BMC Med Educ.

[B10] The Higher Education Commission of Pakistan National Digital Library Programme. http://hec.gov.pk/ereforms/Digital_Libraries.html.

[B11] Kasi PM, Khawar T, Khan FH, Kiani JG, Khan UZ, Khan HM, Khuwaja UB, Rahim M (2007). Studying the association between postgraduate trainees' work hours, stress and the use of maladaptive coping strategies. J Ayub Med Coll Abbottabad.

[B12] Kasi PM, Kassi M, Khawar T (2007). Excessive work hours of physicians in training: maladaptive coping strategies. PLoS Med.

[B13] Hyder AA, Akhter T, Qayyum A (2003). Capacity development for health research in Pakistan: the effects of doctoral training. Health Policy Plan.

